# Leptospirosis in the Asia Pacific region

**DOI:** 10.1186/1471-2334-9-147

**Published:** 2009-09-04

**Authors:** Ann Florence B Victoriano, Lee D Smythe, Nina Gloriani-Barzaga, Lolita L Cavinta, Takeshi Kasai, Khanchit Limpakarnjanarat, Bee Lee Ong, Gyanendra Gongal, Julie Hall, Caroline Anne Coulombe, Yasutake Yanagihara, Shin-ichi Yoshida, Ben Adler

**Affiliations:** 1 Department of Medical Microbiology, University of the Philippines, College of Public Health, Manila, Philippines; 2 WHO/FAO/OIE Collaborating Centre for Reference & Research on Leptospirosis, Queensland Health Forensic and Scientific Services, Brisbane, Australia; 3 Western Pacific Regional Office, World Health Organization, Manila, Philippines; 4 South East Regional Office, World Health Organization, India; 5 Department of Bacteriology, Kyushu University, Fukuoka, Japan; 6 Australian Research Council Centre of Excellence in Structural and Functional Microbial Genomics, Department of Microbiology, Monash University, Melbourne, Australia

## Abstract

**Background:**

Leptospirosis is a worldwide zoonotic infection that has been recognized for decades, but the problem of the disease has not been fully addressed, particularly in resource-poor, developing countries, where the major burden of the disease occurs. This paper presents an overview of the current situation of leptospirosis in the region. It describes the current trends in the epidemiology of leptospirosis, the existing surveillance systems, and presents the existing prevention and control programs in the Asia Pacific region.

**Methods:**

Data on leptospirosis in each member country were sought from official national organizations, international public health organizations, online articles and the scientific literature. Papers were reviewed and relevant data were extracted.

**Results:**

Leptospirosis is highly prevalent in the Asia Pacific region. Infections in developed countries arise mainly from occupational exposure, travel to endemic areas, recreational activities, or importation of domestic and wild animals, whereas outbreaks in developing countries are most frequently related to normal daily activities, over-crowding, poor sanitation and climatic conditions.

**Conclusion:**

In the Asia Pacific region, predominantly in developing countries, leptospirosis is largely a water-borne disease. Unless interventions to minimize exposure are aggressively implemented, the current global climate change will further aggravate the extent of the disease problem. Although trends indicate successful control of leptospirosis in some areas, there is no clear evidence that the disease has decreased in the last decade. The efficiency of surveillance systems and data collection varies significantly among the countries and areas within the region, leading to incomplete information in some instances. Thus, an accurate reflection of the true burden of the disease remains unknown.

## Background

Leptospirosis is a globally important zoonotic disease [1-3], most commonly found in tropical or sub-tropical countries and may be prevalent in both urban and rural settings. Annual incidence is estimated from 0.1-1 per 100,000 in temperate climates to 10-100 per 100,000 in the humid tropics. A disease incidence of more than 100 per 100,000 is encountered during outbreaks and in high-exposure risk groups [4]. Worldwide prevalence rates remain underestimated [4,5], most likely as a result of the protean nature of the disease.

Leptospirosis is caused by spirochetes of the genus *Leptospira*. Leptospires were first identified as the cause of Weil's disease in Japan, where it was common among coal miners [6]. The genus *Leptospira *contains at least 18 species classified on the basis of DNA relatedness and more than 300 serovars based on agglutinating LPS antigens. Approximately half of the pathogenic serovars belong to *L interrogans *or *L. borgpetersenii*. Saprophytic species such as *L. biflexa *occur in the environment, but play no role in disease.

Rodents and domestic mammals, such as cattle, pigs and dogs, serve as major reservoir hosts [6-8], but *Leptospira *has been isolated from virtually all mammalian species. Infected animals may excrete leptospires intermittently or regularly for months or years, or for their lifetime [6]. Vaccinated animals may still shed infectious organisms in the urine [6].

**Figure 1:**
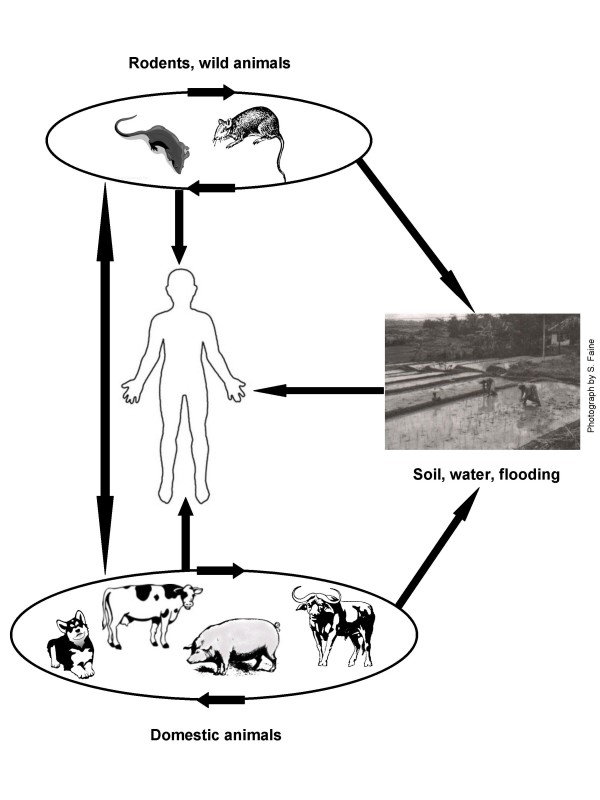
**Epidemiology of leptospirosis**. Carrier animals, domestic or wild, cycle leptospires within the population. Leptospires may then be transmitted to humans directly by contact with infected urine or indirectly via contaminated soil or water, especially in times of flood. Human leptospirosis constitutes a dead-end infection; human to human transmission is virtually unknown.

Human infection results from direct or indirect exposure to the urine of carrier animals. Leptospires gain entry into the blood stream via cuts, skin abrasions or mucous membranes. Leptospirosis has often been considered as an occupational disease, but recreational activities and traveling in endemic countries are also recognized as risk factors [8,9]. Significant exposure also occurs from normal daily activities, with high rates of infection during heavy rainfall and flooding [7,10,11]. Urban slum dwellers in areas with poor sanitation are at particularly high risk [7]. The epidemiology of leptospirosis is illustrated in Figure 1.

The spectrum of clinical presentations of human leptospirosis ranges from asymptomatic to fatal. The majority of infections is subclinical or results in mild self-limiting systemic illness [10]. However, the case fatality rate in severe leptospirosis may be as high as 20% [10]. A particular problem is that that leptospirosis can be misdiagnosed, due to its wide spectrum of symptoms which may mimic the clinical signs of many other diseases, such as dengue fever, hantavirus infection and malaria.

This review describes the current trends in the epidemiology of leptospirosis in the Asia Pacific region, the existing surveillance systems for leptospirosis in these regions, and presents the existing prevention and control programs in the region. Data on the prevalence of leptospirosis in each country were sought from official national organizations, international public health organizations, online articles and the scientific literature. However, the efficiency of surveillance systems and data collection varies significantly among the countries and areas within the region, leading to incomplete information in some instances.

## Methods

Data were obtained from official national organizations, international public health organizations, personal communications, MEDLINE, online articles and the scientific literature. Papers were reviewed and relevant data were extracted.

## Results and discussion

### I. Surveillance Systems

Surveillance of leptospirosis has proven to be an effective public health tool in preventing large outbreaks of the disease [12]. Whereas effective surveillance systems with appropriate laboratory support exist in developed countries in the Asia Pacific region, they are generally lacking in the disease-endemic, developing countries. Even when present, the systems may not meet acceptable international quality assurance standards. Despite the existence of the WHO case definition for leptospirosis, the infrequent use of confirmatory laboratory testing, and the inability to link clinical, epidemiologic, and laboratory data have hindered system utility [9].

A functional disease surveillance system, for both humans and animals, is essential for the effective control of leptospirosis. In addition to a number of research institutions, there are three WHO collaborating centers for leptospirosis in the region: the WHO/FAO/OIE Collaborating Centre for Reference and Research on Leptospirosis, Australia and Western Pacific Region based in Brisbane, Australia, and the Regional Medical Research Center in Port Blair, India.

### II. Epidemiology

The actual incidence of leptospirosis in the Asia Pacific region is not well-documented, similar to the situation in many regions worldwide. Aside from underreporting, incidence data are further compromised by the unavailability of laboratory diagnosis [10]. Within the Asia Pacific region, Southeast Asia and Oceania appear to have the highest incidence of leptospirosis [11].

Major outbreaks of the disease in South-East Asia due to flooding were reported in Orrisa (1999), Jakarta (2003) and Mumbai (2005). It has been a continuing and significant problem in the densely populated, flood-prone low lying areas of India.

In **India**, the disease has been found more commonly associated with natural disasters especially during the monsoon period at which times acute epidemics may eventuate [13]. A multi-centric study in India showed that leptospirosis accounts for about 12.7% of cases of acute febrile illness responsible for attendance at hospitals [14]. Carrier animals include rats, pigs, cattle, bandicoots and dogs. The predominant serovars are Copenhageni, Autumnalis, Pyrogenes, Grippotyphosa, Canicola, Australis, Javanica, Sejroe, Louisiana, and Pomona. Outbreaks of leptospirosis have increasingly been reported from Kerala, Gujarat, Tamil Nadu and Karnataka and sporadic cases have been reported from Goa, Andhra Pradesh and Assam [13].

Leptospirosis has been known to be endemic since the early part of 20^th ^century on the Andaman and Nicobar Islands, where serovars Ratnapura, Valbuzzi and Grippotyphosa have been recently documented as causes of severe epidemics [15]. Highest rates occur during October to November, with seroprevalence of up to 55% in the general population [8]. Interestingly, the predominance of leptospirosis in coastal regions is most likely correlated with the presence of semi-domestic brown rats. In the inland urban regions, other serovars with other host animals/rodents were presumed to cause the 'mild' leptospirosis that is usually unrecognized or misdiagnosed. **The Maldives **reported their first confirmed case of human leptospirosis in November 2000. Since then, the disease is under national surveillance (Geela Ali-personal communication. Director, Center for Community Health and Disease Control, Maldives).

In **Bangladesh **and **Nepal**, countries with similar environmental and sanitary conditions to those in India, the problem of overcrowding also contributes to the burden of disease [16,17]. A serological survey in a rural flood prone district of Bangladesh in 1994 showed 38% seropositivity in 89 samples of human sera tested, indicating that the rural population is at high risk of leptospiral infection [18]. In 2000, acute-phase serum specimens from 359 dengue-negative patients in Dhaka were assessed for leptospirosis; *Leptospira *was detected by PCR in 63 (18%) of them. Poverty and poor education were implicated as conditions leading to rodent-borne transmissions [16]. A number of serological studies carried out in Nepal during the last decade showed the presence of antibodies against major *Leptospira *spp. prevalent in Asia. [17,19,20]. There are no published human leptospirosis data for **Bhutan**. However, suspected leptospirosis human cases have been reported from Chukha district during summer season after flooding in 2008. Cases commonly present with a history of fever for 10 days and autopsy reports indicate organ failure or pulmonary hemorrhage as cause of death. The National Center for Animal Health has detected leptospirosis cases among pigs based on positive serology. (Karma Lhazeen - personal communication. Programme Officer, Vector-borne Disease Control Programme, Gelephu, Bhutan)

Increasing numbers of leptospirosis cases have been observed over recent years in **Sri Lanka **despite the implementation of a set of strategies for its control and prevention. It has a reported annual incidence of approximately 14/100,000. Over 19 leptospiral serovars belonging to over 7 serogroups have been isolated and incriminated as the causative agents of leptospirosis in humans and/or animals throughout the country [21]. Peak incidence is associated with the rice paddy-harvesting seasons wherein an increase in the rodent population in and around the fields is observed. The majority of patients [43.5%] had been exposed in the paddy fields, indicating occupational exposure among the farmers [21]. In 2007, a total of 2195 cases was reported, 40% more than in the previous year. An unusually high case fatality rate and high reporting were noted in districts not previously identified as high risk areas [22]. This alarming trend has cautioned the Ministry of Health to re-assess its on-going strategies on leptospirosis.

In **Thailand**, data from disease notification reports indicated an increase in the incidence rate from less than 0.3 per 100,000 in 1995 to 23.7 in 2000, with a drop in subsequent years. The vast majority of the cases [90%] was reported in the Northeast region, primarily as a result of flooding [23] and the emergence of a highly virulent clone [24]. A recent seroprevalence study at a hospital on the Thai-Myanmar border revealed that 17% of patients who sought treatment for fever were diagnosed with leptospirosis [25]. Most infections occur in agricultural workers, primarily rice producers, whose bare-footed activity during work in rice fields constitutes the main risk. Outbreaks of leptospirosis correspond with the rainy season, with an increase in cases beginning in August and decreasing in November. In **Myanmar**, veterinary investigations have reported the presence of leptospirosis in the animal population [26] while data for human leptospiral cases are not available.

Leptospirosis probably poses a severe and highly underestimated continuous health problem in **Indonesia**. In 2001, 139 human serum samples were tested out of which 18.7% were positive, indicating predominantly presumptive serovar Bataviae infections [27]. In the wake of massive flooding in Indonesia in January 2002, a leptospirosis outbreak occurred, notably in the capital Jakarta on Java. In this outbreak, 12.0% out of 418 samples were seropositive against serovars Bataviae or Hardjo [27]. During this outbreak, high seropositivity rates were detected among potential domestic infection reservoirs such as cats, dogs and cattle. There has been a nationwide increase in the number of reported human cases since 2006. Out of 667 reported human cases in 2007, 93% were laboratory confirmed. The case fatality rate was 8%. In the first quarter of 2008, 269 cases have been detected [Asri Mohammad - personal communication. WHO country office, Indonesia].

A suspected clinical case of leptospirosis was recorded during the flooding season in **Timor-Leste **in 2006 but it could not be confirmed [Sidartha Yuwono - Personal communication. WHO country office, Timor-Leste].

For the year 2008, the total number of notifications for **Australia **was 112 cases [28]. This is the lowest number of reported cases in over ten years and represents a downward trend since a peak in 1999. This reduction has been attributed to persistent drought conditions over the last few years. The northern state of Queensland accounts for most of the leptospirosis cases in the country [71.2%], with serovars Zanoni, Australis and Hardjo accounting for 58.9% of these. The disease has been mainly an occupational hazard of livestock and agricultural workers. Serovars Arborea and Topaz have recently emerged as significant causes of human leptospirosis. In the southern temperate climate states, serovar Hardjo acquired from cattle constitutes the main infecting serovar.

Leptospirosis is the most common occupationally acquired infectious disease in **New Zealand**. The great majority of cases of leptospirosis occurs among livestock farm workers and meat processing workers [29]. An assessment of human leptospiral epidemiology in New Zealand, conducted by Thornley et al. [30] from 1990 to1998, showed an annual incidence of 4.4 per 100,000 population. However, the analysis of trends over this 9-year period revealed that a changing epidemiological pattern was evident, with the emergence of *L. borgpetersenii *serovar Ballum as a more frequent cause of human infection.

The **Philippines **faces a serious problem of leptospirosis. Poor sanitation and the increase in urban slums along with frequent typhoons and expansion of flooding areas in the country have exacerbated the risk of infection [31]. Information on the prevalent *Leptospira *serovars among humans in the Philippines dates back to the late 1960s and 1970s, when antibodies against serovars Pyrogenes, Bataviae, Pomona, Grippotyphosa, Manilae and Javanica were detected among high-risk workers such as abattoir employees, dog pound workers and fish inspectors in Manila. Outbreaks of leptospirosis due to flooding were reported in prisons, penal farms and in many parts of the country [32,33]. Yanagihara et al. [7] performed a seroepidemiological survey from 1998 to 2001, showing that 70% of suspected leptospirosis patients (1,200) were seropositive. They also isolated leptospires from humans and rats (both house and field) in metropolitan Manila and nearby provinces, and also in the city of Iloilo. In 1999, the Department of Health listed leptospirosis as a notifiable disease in the country.

In **Japan**, the number of leptospirosis cases had increased, reaching more than 200 reported deaths annually until 1960 [34,35], mostly in rice-field farmers. Since then, this number has decreased rapidly, primarily due to the mechanization of agriculture in this country, farmers' use of rubber boots when working in the fields and the use of an inactivated vaccine [7,10]. Currently, less than 20 human cases are reported annually, mostly contracted during recreational activities or imported from Southeast Asian countries, or by contact with imported infected animals [7,36]. Serovars Icterohaemorrhagiae, Copenhageni, Autumnalis, Hebdomadis and Australis have been recognized as major causes of human leptospirosis. Most current cases are confined to the southernmost islands, the Okinawa archipelago [7,36].

The first leptospiral case in **Korea **was documented in 1984, and since then, it has become recognized as a major public health problem [37]. Serovars Lai, Copenhageni and Canicola were isolated from 1984-1990 [38]. A leptospiral vaccination programme was initiated in 1988, targeting high risk groups in the rural areas and may have been responsible for the reduction of cases in the succeeding years. However, during the last decade, cases have been increasing slightly. In the year 2007, 208 cases of human leptospirosis were recorded. The bulk of cases consisted predominantly of rice field farmers, occurred in the rural areas of Jeonnam and Jeonbuk (Sang-Hee Park - personal communication, NIH, Korea Center for Disease Control & Prevention. [39]).

To date, 37 serovars from humans and animals have been isolated in **Malaysia **[40]. Human infections in the 1950s, depicted as febrile cases, were commonly observed in military personnel and civilians [40]. Succeeding investigations revealed that the disease is an occupational hazard mostly for people engaged in agricultural and livestock activities, such as cattle farmers, veterinary staff, tin miners, farmers and paddy planters [40]. Recent studies reported a high enzootic incidence in the animal population [40,41]. In 2000, a recreational event-related outbreak of leptospirosis originated in Malaysia [42].

**Vietnam**, **Cambodia **and **Laos **have been considered endemic for leptospirosis. In 2002, a study by Laras and colleagues [43] confirmed the endemicity of the disease in many areas in these countries. In this study, Hurstbridge, Bataviae and Icterohaemorrhagiae were reported to be the predominant serovars identified in patients with clinical jaundice. Serovar Bataviae, on the other hand, was most frequently isolated from patients with non-malarial febrile illness. Pyrogenes and Hurstbridge were the principal serogroups identified among patients with hemorrhagic fever. Recent seroprevalence studies conducted in flood-prone rural areas in Laos [44] and in southern Vietnamese children [45] showed an overall seroprevalence of 23.9% and 12.8%, respectively. In a human seroprevalence study conducted in the Mekong Delta of Vietnam (1998), Bataviae was identified as the most common serogroup [46]. Meanwhile, the highest seroprevalence rate among sows in the same area was recorded with serovar Bratislava [47]. A study in Takeo province, Cambodia estimated an annual incidence of 7.65 per 100,000 population [48] with Javanica and Australis as the main serogroups.

Leptospirosis has been notifiable since 1955 in **People's Republic of China**. Accumulated evidence from 1955-1993 showed an annual incidence of 7.1 per 100,000 with a mortality rate of 1.0% in 26 provinces across the mainland [Gu J-W, Jiang X-G, Guo X-K, unpublished]. Cases were concentrated in July to October, particularly in August and September. Icterohaemorrhagiae and Pomona are the dominant serogroups frequently associated with leptospirosis in the country [Gu J-W, Jiang X-G, Guo X-K, unpublished]. From January 2002 to October 2007, the Ministry of Health reported about 1,500 confirmed cases and 50 deaths in mainland China [49]. There is a low incidence of leptospirosis in **Hong Kong SAR**. There was only 1 local case in 2001, 2 local cases in 2002, 1 imported case in 2003 and 1 imported and 5 local cases in 2004, 7 in 2005 and 1 in 2006 [50]. Although no published reports on human leptospirosis in **Mongolia **can be found, the disease is most likely endemic, as numerous seroprevalence studies indicate a high incidence of animal leptospirosis [51].

In a regional human leptospirosis survey conducted in the **Western Pacific Islands **[52], among 263 cases of suspected leptospirosis cases from September 2003 to December 2005, 69 were confirmed from 7 islands: Futuna, Raiatea and the Marquesas Islands, where outbreaks were apparent, and Vanuatu, Fiji, Palau and Wallis where sporadic cases indicated the presence of the disease. Icterohaemorrhagiae and Australis were the dominant presumptive serogroups, indicating linkage to a rodent reservoir. The rate of exposure has more often been related to normal daily activities than following a specific occupational exposure. According to World Organisation for Animal Health (OIE) data, the disease is enzootic in **French Polynesia **and **New Caledonia**. In a retrospective study of 192 cases of human leptospirosis in New Caledonia from 1989 to 1993, the annual incidence rate was estimated to be 30 per 100,000 population [53]. Serovar Icterohaemorrhagiae was the most prominent among the forty isolates obtained. The annual incidence between 2002 and 2008 has varied from 6.1 to 92 per 100,000, with a large increase in incidence in 2008 (92/100,000) and with signs that this will increase further in 2009. Notifications up to 8 June 2009 were 134, which would equate to an overall figure of over 150/100,000 if the trend continues for the remainder of the year.

Leptospirosis has been considered endemic in the **Commonwealth of the Northern Mariana Islands **[54], although its actual incidence is unknown. Serological evidence in **Palau **indicates that up to 8 percent of "viral syndrome" cases (fever of unknown origin in an adult) were related to acute leptospirosis [55].**American Samoa **reported its first laboratory-confirmed human case in April 2003. A year later, a seroprevalence survey was conducted, showing that 17% of the enrolled adults have had prior infection, with Bratislava as the dominant serovar [56]. Leptospirosis in **Papua New Guinea **dates back to the 1970s; however, data collection on the disease is scarce. A recent investigation in livestock found a dominance of serovar Hardjo [57].

The incidence of leptospirosis in the region is summarized in Table 1.

**Table 1:** Summary of incidence of leptospirosis in the Asia Pacific region.

Annual incidence per 100,000	Country/region
High (>10)	Bangladesh^a^
	Cambodia^a^
	Fiji^a^
	French Polynesia^a^
	India (Andaman and Nicobar Islands)
	Laos^a^
	Nepal^a^
	New Caledonia
	Sri Lanka
	Thailand
	Vietnam^a^
	Wallis and Futuna^a^

Moderate (1 to 10)	American Samoa^a^
	China
	India (mainland)
	Indonesia
	Malaysia
	New Zealand
	Palau^a^
	Philippines
	Marshall Islands^a^
	Vanuatu^a^
	Mongolia^a^

Low (<1)	Australia
	Hong Kong SAR
	Japan
	South Korea
	Taiwan

Insufficient information	Bhutan
	Myanmar
	North Korea
	Papua New Guinea
	Timor-Leste
	Western Pacific islands not mentioned above

^a ^based on unofficial information where official data are not available

### III. Prevention and Control

Leptospirosis is a preventable disease. Control measures must include risk communications, improvement in sanitation and living conditions, and rodent control, as well as both prophylactic and therapeutic medical and veterinary interventions.

#### a. Social control measures

Social control measures are an essential component of the success of prevention and control of leptospirosis. Effective risk communication strategies will ensure that appropriate approaches are used with identified at-risk groups, but also with the groups that can influence the knowledge and practices of these at-risk groups. Such approaches include awareness, health promotion and health education, advocacy and capacity building.

Leptospirosis has typically been considered an occupational disease and thus social control measures directed towards agriculture and other at-risk workers are critical. For instance, a study suggested that the use of trousers and long skirts among pond cleaners in Thailand is a strong protective factor against infection and that further education efforts on protective practices are needed [58]. Awareness of the disease, particularly its protean manifestations, and education about preventative measures with labor organizations and employers should also be implemented. Intensive, well-directed education and publicity campaigns in New Zealand, used in conjunction with a campaign for immunization of cattle, were successful in reducing the incidence of leptospirosis [29,30,36].

In urban slums with high levels of poverty, environmental sources of transmission include open refuse deposits and animal reservoirs, open sewers and flooding; infection is thus associated with exposure in and around the household [58]. Several approaches are needed to reduce risk factors in these situations. Health education campaigns targeted at household occupants as well as advocacy on social determinants of health and concrete actions to reduce health inequity are also needed. Moreover, awareness and education are necessary among administrative, education and health professionals in human and veterinary medicine including primary health care workers, wild life and conservation scientists and infrastructure and urban planners [6].

#### b. Rodent control

Rodents are recognized as the most important reservoirs in the transmission of leptospirosis [3,10], especially of more severe forms. Rodent-vector control activities must consider the epidemiological implications, ecology, and dynamic population of the rodents [59]. Sample collection of rodent species as well as documentation of their infestation levels is required [60]. The use of rodenticides, entrapment of animals, and improved sanitation have been shown to successfully diminish the risk of leptospirosis transmission [60,61]. In India, the timing of rodent control was shown to be a vital consideration in the prevention of disease transmission. The rodent breeding period starts with the southwest monsoon [60], suggesting that rodent control measures in the pre-monsoon period would bring better vector control.

#### c. Vaccination

On-going research into numerous vaccine preparations for humans and animals is well documented. This includes the use of inactivated and attenuated vaccines, recombinant protein or lipoprotein vaccines, LPS vaccines and DNA vaccines [62,63].

The local variability in serovars of endemic leptospiral strains complicates the development of a vaccine that could be used worldwide [6,10]. Current bacterin vaccines elicit immunity that is generally restricted to serovars with related agglutinating LPS antigens. In the region, human vaccines against *Leptospira *are available in China, Japan, Korea and Vietnam (Sang-Hee Park - personal communication, NIH, Korea Center for Disease Control & Prevention. [64]), whereas commercial *Leptospira *vaccines for animals are available in many countries [62,64]. Although treatment and vaccination of livestock are not mandatory, low sporadic occurrence of the disease in these animals was observed in endemic countries after initiation of a vaccination program [6,65]. Vaccination of domestic animals may be considered too expensive in developing countries, and, in any event, may not prevent leptospiruria in vaccinated animals [6]. Clearly, vaccination is not possible for non-domestic reservoir animals.

#### d. Chemoprophylaxis

Use of doxycycline as a prophylactic measure has been reported [6], but is clearly only suitable in instances where exposure can be predicted accurately and is for a short period of time. It is not appropriate as a routine, long term, community-based practice.

## Conclusion

Leptospirosis remains a major endemic environmental disease in most countries in the Asia Pacific Region. Socio-economic conditions, population density, climatic and environmental conditions, and behavioral and occupational habits of humans are determinants of the incidence and prevalence of the disease. The ability of all countries in the region to accurately report and monitor leptospirosis hinges strongly on their respective capacity to provide accurate and reliable diagnostic and reporting services.

Leptospirosis is preventable. Host/reservoir control measures, environmental control programs and animal vaccination, in conjunction with a strong surveillance system may significantly reduce, if not eliminate, the disease. Thus, an effective collaboration among countries in the region is the key to successfully combating this public health problem.

## Competing interests

The authors declare that they have no competing interests.

## Authors' contributions

AV, NB, TK, BO, SY and BA conceived of the study and participated in its design and coordination. All authors (AV, LS, NB, LC, TK, KL, BO, GG, JH, CC, YY, SY and BA) contributed to the review of studies and interpretation of data. AV, LS, NB, BO, JH, CC and BA drafted the manuscript and prepared the final version for submission. All authors read and approved the final manuscript.

## Pre-publication history

The pre-publication history for this paper can be accessed here:

http://www.biomedcentral.com/1471-2334/9/147/prepub
